# The neural underpinnings of facial emotion recognition in ischemic stroke patients

**DOI:** 10.1111/jnp.12240

**Published:** 2021-02-08

**Authors:** Nils S. van den Berg, Edward H. F. de Haan, Rients B. Huitema, Jacoba M. Spikman, the visual brain group, Frank Erik de Leeuw, Gert Jan Luijckx, Matthijs A.H.L.L. Raemaekers, Anouk R Smits, Ben A Schmand, H Steven Scholte, Jacoba M Spikman, L Jaap Kappelle, Linda Geerligs, Martine J.E. van Zandvoort, Matthan W.A. Caan, Mathias Prokop, Nick F Ramsey, Nikki A Lammers, Noor Seijdel, Paul J Nederkoorn, Bob Kentridge, Roy P.C. Kessels, Selma Lugtmeijer, Yair Pinto

**Affiliations:** ^1^ Department of Psychology University of Amsterdam The Netherlands; ^2^ Department of Neurology University Medical Center Groningen University of Groningen The Netherlands

**Keywords:** basic emotions, cerebrovascular accident, facial emotion recognition, ischaemic stroke, voxel‐based lesion–symptom mapping

## Abstract

Deficits in facial emotion recognition occur frequently after stroke, with adverse social and behavioural consequences. The aim of this study was to investigate the neural underpinnings of the recognition of emotional expressions, in particular of the distinct basic emotions (anger, disgust, fear, happiness, sadness and surprise). A group of 110 ischaemic stroke patients with lesions in (sub)cortical areas of the cerebrum was included. Emotion recognition was assessed with the Ekman 60 Faces Test of the FEEST. Patient data were compared to data of 162 matched healthy controls (HC’s). For the patients, whole brain voxel‐based lesion–symptom mapping (VLSM) on 3‐Tesla MRI images was performed. Results showed that patients performed significantly worse than HC’s on both overall recognition of emotions, and specifically of disgust, fear, sadness and surprise. VLSM showed significant lesion–symptom associations for FEEST total in the right fronto‐temporal region. Additionally, VLSM for the distinct emotions showed, apart from overlapping brain regions (insula, putamen and Rolandic operculum), also regions related to specific emotions. These were: middle and superior temporal gyrus (anger); caudate nucleus (disgust); superior corona radiate white matter tract, superior longitudinal fasciculus and middle frontal gyrus (happiness) and inferior frontal gyrus (sadness). Our findings help in understanding how lesions in specific brain regions can selectively affect the recognition of the basic emotions.

## Background

Impairments in social cognition are a frequent complication after stroke (Yuvaraj, Murugappan, Norlinah, Sundaraj, & Khairiyah, 2013). Social cognition comprises the cognitive and emotional functions which enable us to process social information and to behave adequately in social situations (Adolphs, 2009). It is a broad construct, consisting of different aspects, including the recognition of socially important information (such as emotional facial expressions), the understanding of behaviour of others (such as creating a Theory of Mind), and empathic behaviour (Blair, 2003). Amongst these different aspects of social cognition, impairments in particularly the ability to recognize emotional expressions have been found to be related to a restricted social participation, a reduced quality of life (Cooper et al., 2014) and to behavioural problems after stroke (Nijsse, Spikman, Visser‐Meily, de Kort, & van Heugten, 2019).

Previous studies have identified a broad network of brain structures that regulate the visuoperceptual and semantic processes necessary for adequate emotion recognition, both at a limbic, subcortical and cortical level (Adolphs, 2002; Borod, 2000; Yuvaraj et al., 2013). These structures roughly involve the orbitofrontal and occipito‐temporal circuits. Not surprisingly, impairments in emotion recognition have been found in a number of patient groups with damage to these brain structures, including patients with stroke (Nijsse et al., 2019; van den Berg, Huitema, Spikman, Luijckx, & de Haan, 2020; Yuvaraj et al., 2013), but also patients with brain tumours (Pertz, Okoniewski, Schlegel, & Thoma, 2020), Traumatic Brain Injury (TBI; Babbage et al., 2011; Spikman et al., 2013; Young, Newcombe, De Haan, Small, & Hay, 1993), subarachnoid haemorrhage (Buunk et al., 2017) and patients with neurodegenerative diseases (NDD), such as Alzheimer’s Disease, Parkinson’s Disease and Fronto‐temporal dementia (Christidi, Migliaccio, Santamaría‐García, Santangelo, & Trojsi, 2018).

Although there is accumulating evidence that impairments in emotion recognition occur frequently after brain injury, studies have presented conflicting results regarding the responsible lesion location. Some studies in patients with non‐progressive lesions have shown that damage in particularly the right hemisphere can underlie impaired emotion recognition (Adolphs, Damasio, Tranel, Cooper, & Damasio, 2000; Philippi, Mehta, Grabowski, Adolphs, & Rudrauf, 2009; Yuvaraj et al., 2013), while other studies did not find support for a lateralization effect (e.g. the study of Campanella, Shallice, Ius, Fabbro, and Skrap (2014) with brain tumour patients). In addition, other studies have indicated a differential contribution of each hemisphere in the ability to recognize either negative or positive emotional expressions, but even within this ‘valence hypothesis’, mixed results have been found. For instance, with regard to impaired recognition of negative emotions, Mattavelli et al. (2019) found a significant relation with damage to the *right* hemisphere in brain tumour patients, whereas Tsuchida and Fellows (2012) found a significant relation with *left* hemispheric damage in patients with focal, frontal lesions with diverse aetiologies.

In addition, it is still unclear whether there is a single brain network responsible for the ability to perceive emotional expressions in general or whether there are separate neural networks involved in identifying separate basic emotions. The existence of a general emotion perception network was suggested in a meta‐analysis (Lindquist, Wager, Kober, Bliss‐Moreau, & Barrett, 2012), while two other meta‐analyses emphasized the existence of distinct networks of the basic emotions (Fusar‐Poli et al., 2009; Vytal & Hamann, 2010). All these meta‐analyses were based mainly on fMRI studies with healthy controls. Most patient studies mentioned in the previous paragraph did not take the specific neural underpinnings of the distinct basic emotions into account. The only lesion–symptom mapping study to date in which distinct emotions were investigated was carried out in patients with brain tumours. The authors found separable regions responsible for the ability to perceive surprised (inferior fronto‐occipital fasciculus and middle frontal gyrus) or happy (fronto‐temporo‐insular region) faces (Mattavelli et al., 2019).

Investigating the basic emotions separately in addition to overall emotion recognition is important, because previous studies have found that impairments in specific basic emotions can be related to specific behavioural disturbances. For example, impaired recognition of fearful expressions has been found to be related to increased risk‐taking behaviour, in both patients with cerebellar stroke (van den Berg, Reesink, et al., 2020), patients with TBI (Visser‐Keizer, Westerhof‐Evers, Gerritsen, van der Naalt, & Spikman, 2016) and patients with NDD (van den Berg, Reesink, et al., 2020). Furthermore, impairments in the recognition of anger have been found to be related to a decreased self‐awareness in stroke patients (Nijsse et al., 2019). Lastly, an impaired recognition of sad expressions in patients with TBI has been found to be related to behavioural changes, reported by significant others (Spikman et al., 2013).

Therefore, the aim of the current study was to investigate the neural underpinnings of emotion recognition in general as well as of the distinct basic emotions, in a large group of ischaemic stroke patients. Because of the inconsistent results from previous studies regarding the role of lesion location and lateralization, we aimed to asses both left and right hemispheric stroke patients, with lesions in both cortical and subcortical structures in the cerebrum. We performed whole brain voxel‐based lesion–symptom mapping (VLSM; Bates et al., 2003) with 3‐Tesla MRI images. In VLSM, a statistic for each voxel is estimated, allowing a satisfactory spatial precision, without a priori alignment of patients to sub‐groups based on their lesion locations (Rorden & Karnath, 2004; Rorden, Karnath, & Bonilha, 2007). As mentioned earlier, impairments in emotion recognition are frequently found in stroke patients (Nijsse et al., 2019; Yuvaraj et al., 2013). We expect that our findings will contribute to a better understanding of impairments in emotion recognition in stroke patients.

## Materials and methods

### Study design

This study is part of the multi‐centre cohort study aimed at assessing subtle visual deficits in patients with ischaemic stroke. The neuropsychological assessment took place at a University Medical Center in the Netherlands between September 2015 and December 2019. The study was approved by The Local Medical Ethics Review Committee.

### Participants

A group of 110 patients (76 men, 69.1%) with a diagnosis of an ischaemic stroke in (sub)cortical areas of the cerebrum (both left‐hemispheric and right hemispheric) was included in the current study. The diagnosis of ischaemic stroke was made by a neurologist, based on the clinical symptoms and confirmed with a 3‐Tesla MRI scan. Patients with severe pre‐existent structural brain damage, such as widespread white matter alterations or global brain atrophy were not included in the current study. The mean age of the patients was 58.5 years (*SD* = 11.7, range = 20–82) and the mean educational level was 5.3 (*SD* = 1.3, on a 7‐point scale ranging from 1 (primary education only) to 7 (university education)). In total, there were 48 patients with right hemispheric damage, 44 with left hemispheric damage and 18 with bilateral damage. The median lesion size was 6,269 mm^3^. Examination took place between two weeks and six months post‐stroke (mean time interval in days = 59.9, *SD* = 31.1, range = 13–174 days). The neuropsychological assessment and MRI scan took place at the same day in all patients, except for 13 patients. In these patients, the MRI took place in the subacute phase after stroke, but the assessment of the emotion recognition test took place during a later visit with a mean of 20.7 months (*SD* = 3.5, range = 16–26 months) after the MRI scan. Most patients were on antihypertensives and anticoagulant medication. No patients were on antipsychotic medication, three patients were on anticonvulsant medication and eight patients were on anti‐depressant medication. These eight patients had already started this anti‐depressant medication prior to their stroke. None of the patients had a current major depression at the time of the assessment, as this was one of the exclusion criteria.

In addition, HC data from previous studies at the local department of Neurology were available for the emotion recognition task. Data matched for age, educational level and sex were selected from these datasets, resulting in a group of 162 HC’s (109 men, 67.3%) with a mean age of 56.5 years (*SD* = 12.3, range = 26–82) and a mean educational level of 5.5 (*SD* = 1.1). There were no significant differences between patients and HC’s with respect to age (*t* (241) = −1.3, *p* = .18), educational level (*t* (211) = 1.2, *p* = .24), and sex (*χ* ^2^ (1) = 0.1, *p* = .75).

Exclusion criteria for all participants involved serious psychiatric (such as a bipolar disorder) or neurological disorders (other than an ischaemic stroke in case of patients, such as TBI), pre‐existing cognitive decline (IQCODE score > 3.6), substance abuse or inadequate understanding of the Dutch language. All participants signed a written informed consent prior to participation and were treated in accordance with the Declaration of Helsinki.

### Neuropsychological measures

#### Cognitive and mood screening

To investigate the general cognitive status of the patients, patients were assessed with the Dutch version of the National Adult Reading Test (NART) as a measure of pre‐morbid IQ, the Clock‐Drawing Test as an indicator of visuo‐construction (possible score range: 0–14), the Digit Span Test as a measure of working memory span (possible score range: 0–16). The Hospital Anxiety and Depression Scale (HADS) was used as a screening for anxiety (possible score range: 0‐21) and for depressive complaints (possible score range: 0–21).

#### Emotion recognition

The Ekman 60 Faces Test of the Facial Expressions of Emotions – Stimuli and Test (FEEST; Voncken, Timmerman, Spikman, & Huitema, 2018; Young, Perrett, Calder, Sprengelmeyer, & Ekman, 2002) was used to assess facial emotion recognition. In this test, 60 faces expressing one of the basic emotions anger, disgust, fear, happiness, sadness and surprise are presented, each 10 times for 3 s, in a randomized order. Then, six labels are presented on the screen and participants have to indicate which emotional expression is shown. The raw scores of the six basic emotion scores range from 0 to 10 and the raw total score ranges from 0 to 60. Based on the Dutch normative data of the FEEST in which age, educational level and sex into were taken into account (Voncken et al., 2018), percentile scores could be determined for each of the basic emotion scores and the total score, named FEEST Ang, FEEST Disg, FEEST Fear, FEEST Hap, FEEST Sad, FEEST Surp and FEEST Tot.

### MRI

MRI was performed on a Siemens Magnetom Prisma 3‐Tesla MR Scanner at the UMCG and RadboudUMC, and on a Philips R5 3‐Tesla MR scanner at the AmsterdamUMC and the UMCU. For each patient, a 3D‐FLAIR was acquired. The sequence details of the Siemens were: voxel size: 0.9 × 0.9 × 0.9 mm^3^, TI: 1,650 ms, TR: 4,800 ms, TE: 484 ms, FOV: 280 mm. The sequence details of the Philips were: voxel size 1.12 × 1.12 × 0.56 mm^3^_,_ TI: 1,650 ms, TR: 4,800 ms, TE: 253 ms, FOV: 250 mm.

### Data analysis

#### Neuropsychological test analysis

Demographical and neuropsychological measures were compared between patients and healthy controls using *t*‐tests and, in case of not‐normally distributed data, Mann–Whitney U tests were performed. Correlation analyses between variables were performed using Pearson’s correlation and, in case of not‐normally distributed data, Kendall’s tau correlation. All neuropsychological test analyses were performed in IBM SPSS statistics 23. The alpha level was set at .05, two‐sided and Bonferroni–Holm corrections were applied to adjust the alpha levels to control for multiple comparisons.

#### MRI pre‐processing and VLSM

**Figure 1:**
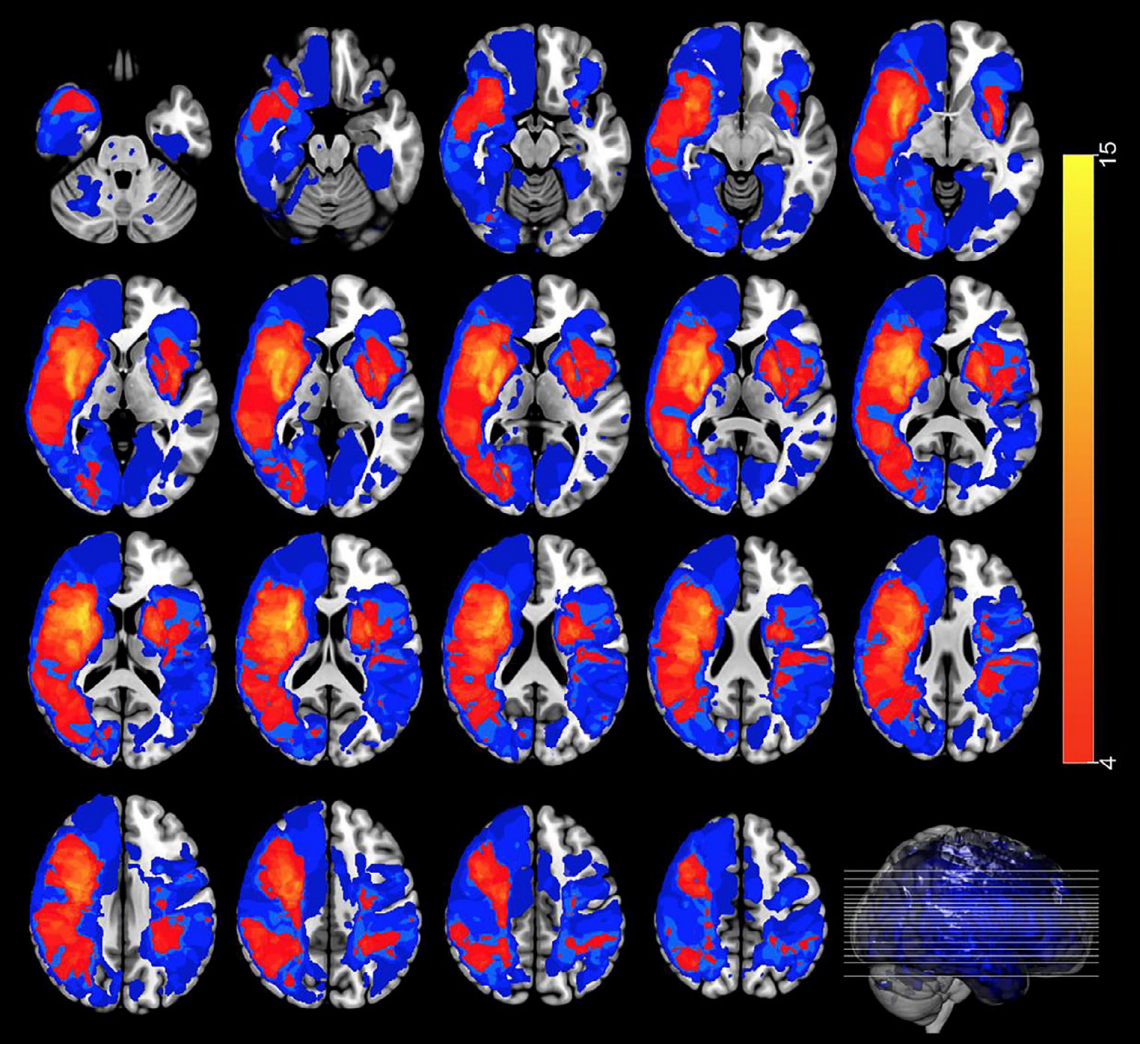
Lesion density plot of the lesions on axial slices. Colours indicate the number of overlapping lesions. Analyses were restricted to voxels in which at least four patients had lesions. Red indicates less lesions and yellow indicates more lesions. Blue indicates less than four lesions in a particular voxel. The left hemisphere is presented on the right.

The semi‐automatic segmentation procedure in ITK‐SNAP (Yushkevich et al., 2006) was used for lesion segmentation. Lesion segmentation was performed by three trained neuroscientists. Each segmentation was checked by another neuroscientist. Cases of uncertainty were additionally discussed with an experienced radiologist or neurologist. To check for the inter‐rater reliability, four scans were randomly selected and lesions in these scans were delineated independently by the three neuroscientists. The mean inter‐rater agreement between all three neuroscientists, measured with the overlap ratio, was 0.76 (Neumann et al., 2009). Subsequently, MRI images and the corresponding lesion maps were normalized with the ‘MR segment‐normalize’ function in the plug‐in clinical toolbox for Statistical Parametric Mapping (SPM12; Rorden, Bonilha, Fridriksson, Bender, & Karnath, 2012) using a unified segmentation–normalization approach (Ashburner & Friston, 2005). In this unified approach, the tissue classification (segmentation), the correction for bias, and the registration (spatial normalization) are combined in a single model (de Haan & Karnath, 2018). To correct for the presence of lesions during normalization, the enantiomorphic correction method (Nachev, Coulthard, Jäger, Kennard, & Husain, 2008) was used in case of unilateral lesions. Cost function masking (CFM; Brett, Leff, Rorden, & Ashburner, 2001) was used in case of symmetrical, bilateral lesions. With enantiomorphic normalization, the lesions are replaced by the intact homologous region from the contralesional hemisphere, while with CFM, the lesions are excluded during the normalization. Therefore, enantiomorphic normalization is the preferred method in case of large lesions, while CFM is more appropriate in case of bilateral, symmetrical lesions (de Haan & Karnath, 2018). A stroke control template based on older healthy participants (mean age of 61.3 years) was used, which has specifically been developed for a stroke population (Rorden et al., 2012). All normalized brains were inspected by comparing the normalized brains and the normalized lesion maps to the template brain using MRIcron (Rorden et al., 2007). All normalized lesion maps were then projected on the template brain. A lesion density plot was then created by the normalized lesion maps (Figure 1).

Voxel‐based lesion–symptom mapping (Bates et al., 2003) was performed using NiiStat (https://github.com/neurolabusc/NiiStat). Voxel‐wise *t*‐tests were performed, comparing the performance on the FEEST between patients with and patients without a lesion in that particular voxel. FEEST percentile scores were used, to control for age, sex and educational level. Analyses were restricted to voxels in which at least four patients had lesions (232,682 voxels) in order to minimize biased estimates of the parameter, based on previous studies (e.g. Dal Cristofori et al., 2019; Monte et al., 2013; Ivanova et al., 2018). Permutation thresholding was used to control for multiple comparisons. Permutation thresholding tries to find whether observed *t*‐test statistics at each voxel can actually be attributed to the status of that voxel: lesioned versus non‐lesioned (de Haan & Karnath, 2018). In total, 5,000 permutations were performed, with a corrected threshold set at *p* = .05. The identification of the significant lesion–symptom associations was performed by using the Automated Anatomical Labeling (AAL) atlas and the Johns Hopkins University (JHU) white matter atlas.

## Results

### Neuropsychological test results

The mean patient scores on the screening tests for cognition and mood disorders are presented in Table 1. The results of Pearson’s correlation analyses between these mean scores on the one hand and FEEST Tot on the other hand are also shown in this table. As can be seen, these correlations were low and, except for NART‐IQ, non‐significant. Although the relationship between NART‐IQ and FEEST Tot was significant, this relationship was weak. Moreover, the NART‐IQ is a *verbal* measure of pre‐morbid IQ, while the FEEST is not an entirely verbal task. Taken together, it seems likely that the confounding influence of possible cognitive or mood disturbances on FEEST performance is limited.

**Table 1:** Performance on the screening tasks with correlation analyses (Pearson’s *r*) with FEEST Tot within the patient group

Screening‐test	Mean score (*SD*)	Pearson's *r* with FEEST Tot (*p‐*value)
NART‐IQ (*n* = 103)	102.1 (15.2)	.30 (.002)*
Clock‐drawing test (*n* = 103)	11.9 (2.0)	.18 (.063)
Digit Span Forward (*n* = 104)	7.9 (1.9)	.16 (.096)
HADS – anxiety (*n* = 100)	3.9 (3.7)	−.11 (.272)
HADS – depression (*n* = 100)	3.2 (2.9)	−.10 (.322)

FEEST = Facial Expressions of Emotions: Stimuli and Test; HADS = Hospital Anxiety and Depression Scale; NART‐IQ = National Adult Reading Test Intelligence Quotient.

* Significant after Bonferroni–Holm correction.

Table 2 shows that patients were significantly worse than HC’s in the overall recognition of emotions. Furthermore, in comparison to HC’s, patients were significantly worse in the recognition of the emotional expressions of disgust, fear, sadness and surprise after the Bonferroni–Holm correction.

**Table 2:** Performance on the FEEST (raw scores) and independent sample *t*‐tests on differences between HC’s and patients, with Cohen’s d effect sizes between groups

Variable	Patients (*n* = 110)	HC's (*n* = 162)	*t‐*value	*p*‐value	Cohen’s *d*
FEEST Anger, *M* (*SD*)	7.5 (2.1)	7.9 (2.0)	1.9	.061	0.2
FEEST Disgust, *M* (*SD*)	6.1 (2.5)	7.7 (2.1)	5.7	**<.001** *	**0.7**
FEEST Fear, *M* (*SD*)	5.3 (2.4)	6.3 (2.5)	3.3	.**001** *	**0.4**
FEEST Happiness, *M* (*SD*)	9.6 (0.9)	9.9 (0.4)	2.3	.026	0.4
FEEST Sadness, *M* (*SD*)	6.1 (2.3)	7.3 (1.8)	4.7	**<.001** *	**0.6**
FEEST Surprise, *M* (*SD*)	8.4 (1.7)	8.8 (1.2)	2.8	.**005** *	**0.3**
FEEST Total, *M* (*SD*)	42.9 (7.4)	48.0 (6.1)	5.9	**<.001** *	**0.8**

FEEST = Facial Expressions of Emotions: Stimuli and Test.

* Significant after Bonferroni–Holm correction.

### Time since stroke

To rule out the effects of the interval between stroke and neuropsychological assessment, secondary analyses were performed. These analyses showed that there were no significant differences in FEEST performance between patients who performed the FEEST at the time of the MRI and the small group of patients (*n* = 13) who performed the FEEST during the later visit (all *p’*s > Bonferroni–Holm corrected alfa). In addition, the relations between time since stroke and all FEEST measures were low and non‐significant: FEEST Ang (τ = −.05, *p* = .44), FEEST Disg (τ = −.00, *p* = .97), FEEST Fear (τ = −.06, *p* = .39), FEEST Hap (τ = .14, *p* = .06), FEEST Sad (τ = −.04, *p* = .53), FEEST Sur (τ = .06, *p* = .38), FEEST Tot (τ = −.03, *p* = .71).

### VLSM results

**Figure 2:**
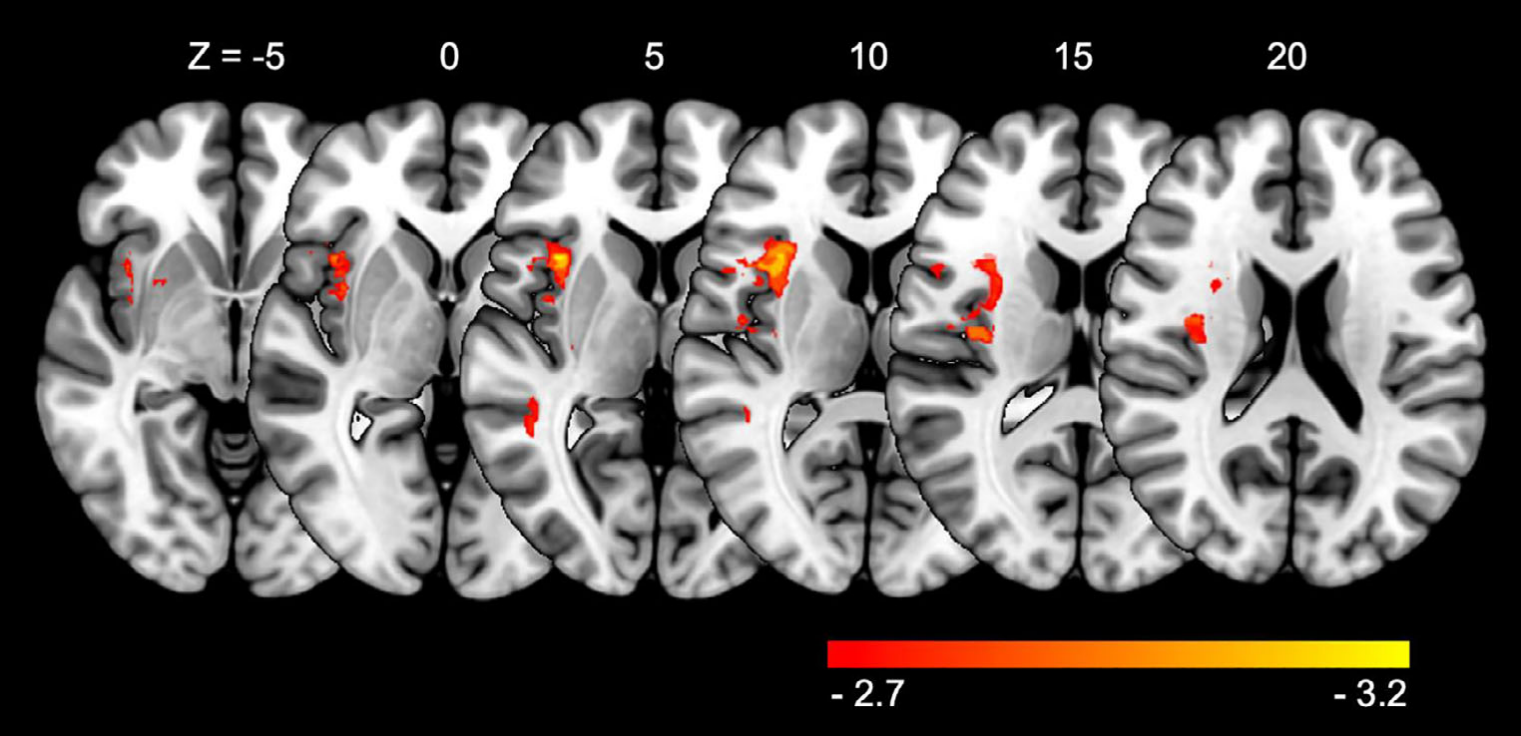
Voxel‐based lesion–symptom mapping results for overall emotion recognition (FEEST Tot). Results are thresholded at a permutation‐derived significance level, with a threshold of *Z* = −2.7. Lighter colours (yellow) indicate increasing test values in comparison to red colours. The right hemisphere is presented on the left.

Figure 2 shows the results of the VLSM analysis for FEEST Tot (*Z*‐scores derived from *t*‐test scores). A significant lesion–symptom association was found for FEEST Tot (*Z* score range: −3.21 to 2.53, *Z* score threshold = −2.71). As can be seen in Figure 2, lesions in particular the fronto‐temporal region in the right hemisphere were related to a lower FEEST Tot. More specifically, significant lesion–symptom relations were found in the insula, caudate nucleus, the lenticular nucleus, putamen, middle frontal gyrus, inferior frontal gyrus (triangular and opercular part), Rolandic operculum and the middle and superior temporal gyrus. The strongest association was found between a lower FEEST Tot and damage to the insula (*Z* = −3.21).

**Figure 3:**
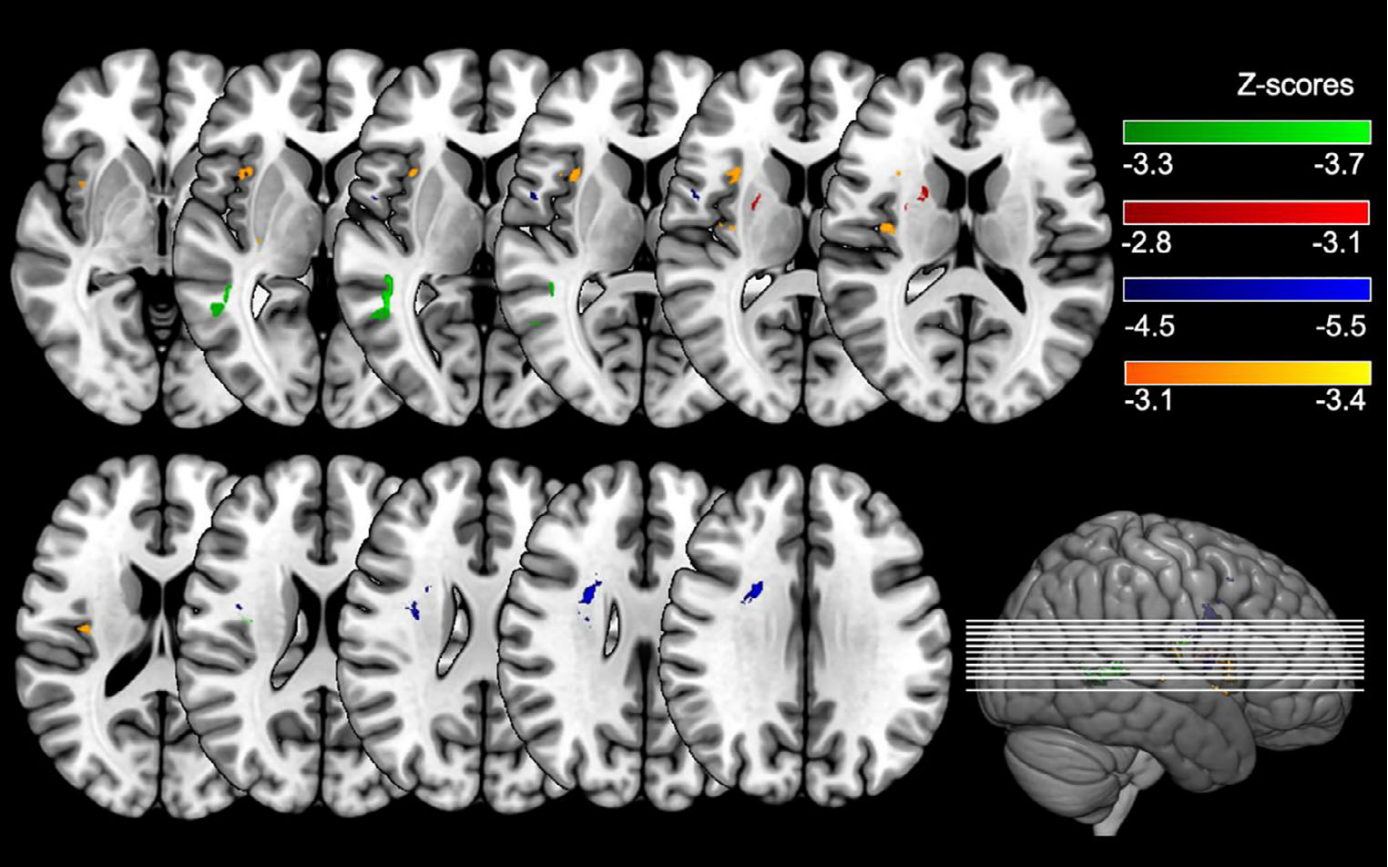
Voxel‐based lesion–symptom mapping results for the ability to recognize anger (green), disgust (red), happiness (blue) and sadness (orange). Results are thresholded at a permutation‐derived significance level, with a threshold of *Z* = −3.3 for anger, *Z* = −2.8 for disgust, *Z* = −4.5 for happiness and *Z* = −3.1 for sadness. Brighter colours indicate increasing test values. The right hemisphere is presented on the left.

Figure 3 shows the results of the VLSM analysis for the basic emotions. As can be seen, significant lesion–symptom correlations were found for FEEST Ang (*Z* score range = −3.66 to 2.29, *Z* score threshold = −3.34), FEEST Disg (*Z* score range = −3.10 to 3.36, *Z* score threshold = −2.84), FEEST Hap (*Z* score range = −5.50 to 1.79, *Z* score threshold = −4.58) and FEEST Sad (*Z* score range = −3.35 to 2.29, *Z* score threshold = −3.19). We did not find significant lesion–symptom correlations for FEEST Fear (*Z* score range = −2.89 to 2.24, *Z* score threshold = −3.29) and FEEST Surp (*Z* score range = −3.00 to 2.30, *Z* score threshold = −3.19).

Interestingly, we found both overlapping brain regions related to the basic emotions, but also separable regions per emotion. Significant overlapping brain regions included the Rolandic operculum (related to FEEST Ang, FEEST Hap and FEEST Sad), the insula (related to FEEST Ang and FEEST Sad) and the putamen (related to FEEST Disg and FEEST Sad). Significant lesions particularly affecting FEEST Ang involved the middle and superior temporal gyrus. Lesions particularly affecting FEEST Disg involved the caudate nucleus. Lesions particularly affecting FEEST Hap involved the superior corona radiate white matter tract, the superior longitudinal fasciculus and the middle frontal gyrus. Lastly, lesions particularly affecting FEEST Sad involved the opercular part of the inferior frontal gyrus. The MNI coordinates of the peak lesion–deficit relationships for all FEEST scores are presented in Table 3.

**Table 3:** Results of the voxel‐based lesion–symptom mapping analysis for the recognition of anger, disgust, happiness, sadness and for the total score on the FEEST

Region	*n* of voxels	MNI coordinates		
		*X*	*Y*	*Z*
Anger				
Middle temporal gyrus	292	46	−41	7
Superior temporal gyrus	57	45	−35	6
Insula	50	36	−10	19
Rolandic operculum	20	44	−10	10
Disgust				
Putamen	37	21	3	14
Caudate nucleus	4	20	6	15
Happiness				
Superior corona radiate	233	24	7	2
Superior longitudinal fasciculus	88	32	−7	24
Rolandic operculum	72	53	3	8
Middle frontal gyrus	8	40	14	50
Sadness				
Insula	493	34	5	9
Putamen	21	30	−18	3
Inferior frontal gyrus	17	38	15	3
Rolandic operculum	3	40	−12	16
FEEST total				
Insula	2,088	36	5	9
Inferior frontal gyrus	437	37	17	3
Putamen	256	30	3	−5
Rolandic operculum	275	40	−6	19
Middle temporal gyrus	73	46	−43	4
Superior temporal gyrus	57	45	−38	7

Note

MNI coordinates in a 3D brain for the peak lesion‐deficit relationships. Locations were labelled using the Automated Anatomical Labelling atlas and the John Hopkins University atlas.

## Discussion

The aim of the current study was to investigate the neural underpinnings of facial emotion recognition, including the brain structures involved in the processing of distinct basic emotions, in a large group of ischaemic stroke patients. The results showed that patients performed significantly worse than healthy controls in the overall recognition of emotional facial expressions, and in particular in the recognition of disgust, fear, sadness and surprise. The VLSM analysis showed that emotion recognition as such was depending on predominantly fronto‐temporal regions in the right hemisphere. With regard to the distinct basic emotions, spatially segregated regions within the right hemisphere were found. Lesions affecting more ventral parts of the temporal region were related to a worse recognition of anger, while lesions affecting more fronto‐temporal areas, including medial and dorsolateral frontal regions and the superior corona radiate white matter tract, were related to a poorer recognition of happiness. A worse perception of disgust was related to damage in the caudate nucleus, while poorer sadness perception was related to damage in the inferior frontal gyrus. Overlapping regions included the insula (related to anger and sadness), Rolandic operculum (related to anger, happiness and sadness) and putamen (related to disgust and sadness).

Our finding that lesions in the right hemisphere were significantly associated with the ability to recognize emotional facial expressions, both positive and negative, is not in line with the ‘valence hypothesis’ (Silberman & Weingartner, 1986; Tucker & Williamson, 1984), as this hypothesis states that damage to the right hemisphere would only be related to difficulties in recognizing negative emotions. Because of the lower lesion coverage in the left hemisphere, we cannot exclude a left hemispheric involvement in emotion recognition. Hence, we cannot confidently conclude that the current results are in line with the right hemisphere hypothesis, which states that particularly the right hemisphere is involved in the recognition of emotions (Blonder, Bowers, & Hellman, 1992; Borod, 2000; Yuvaraj et al., 2013).

Damage to the insula was found to be related to overall worse facial emotion recognition. The insula, a part of the cerebral cortex positioned underneath the frontal, temporal and parietal lobes, has many interconnections with other brain structures, including the prefrontal cortex and subcortical structures, such as the amygdala (Damasio, 2003; Gasquoine, 2014). According to Damasio (2003), emotional experiences are formed in the insula, particularly in the right insular region. Craig (2009) described that the anterior insular cortex was related to both the experience of emotional feelings and to the recognition of emotions. All aspects of emotional processing, including the experience of emotions and the recognition of emotions have been found to be highly interrelated (Enticott, Johnston, Herring, Hoy, & Fitzgerald, 2008; Price & Harmon‐Jones, 2015). Therefore, it seems likely that damage to the insula in our patients has resulted in a disturbance in emotional processing, including in the recognition of emotional expressions.

This is the first VLSM study in a group of stroke patients that investigated the distinct basic emotions in addition to overall emotion recognition. Interestingly, apart from overlapping regions, spatially segregated brain regions for the distinct emotions were found According to Adolphs (2002), the ability to perceive emotional facial expressions depends on adequate visuoperceptual processes as well as on the accurate classification of the emotions. The visuoperceptual processes are expected to be related to more temporo‐occipital parts of the brain, whereas the emotional part is expected to be related to more frontal parts of the brain. Largely consistent with this, we found both fronto‐temporal regions and temporal regions to be related to emotion recognition.

Interestingly, the middle and superior temporal gyrus were exclusively related to the ability to recognize angry expressions. The middle and superior temporal gyrus have previously been related to social cognitive functions in general (Allison, Puce, & McCarthy, 2000; Jou, Minshew, Keshavan, Vitale, & Hardan, 2010). Sprengelmeyer, Rausch, Eysel, and Przuntek (1998) also found, amongst others, the middle temporal gyrus (MTG) to be related to anger perception. However, their study showed that the left MTG was related to anger perception instead of the involvement of the right MTG in the current study. According to Allison et al. (2000), the superior temporal gyrus (STG) region is an important region in the initial perceptual analysis of social information. They suggested that the STG is involved in the integration of both motion‐ and form‐related visual information. Our finding of a relation between impaired anger recognition and damage to this region therefore indicates that especially the ability to perceive anger could depend on more visuoperceptual processes in comparison to some of the other emotions. This idea is furthermore supported by findings of Du and Martinez (2011), who found that negative emotions (anger and sadness), were only correctly recognized at high resolutions, indicating that these negative emotions require more extensive visuoperceptual processes (depending on occipito‐temporal brain areas) in comparison to positive emotions.

Brain regions related to the ability to perceive happy faces involved the superior corona radiate white matter tract, the superior longitudinal fasciculus, Rolandic operculum and the middle frontal gyrus. The involvement of the superior corona radiate white matter tract has previously been found to be related to overall emotion perception (Rigon, Voss, Turkstra, Mutlu, & Duff, 2019). In this study of Rigon et al. (2019), diffusion tensor imaging (DTI) was used to investigate the relationship between white matter integrity and facial emotion recognition. They found, amongst others, the right corona radiate white matter tract to be related to overall emotion perception in a matching task. However, they did not take the distinct basic emotions into account. The current study shows that this white matter tract might especially be involved in the ability to adequately perceive particularly happy faces. Future studies should be performed to further investigate this white matter tract involvement in specific emotion recognition.

A poorer recognition of disgust was related to damage in the caudate nucleus, in addition to damage in the putamen (the latter being also related to a poorer recognition of sadness). The caudate nucleus and putamen form the striatum, which is part of one of the frontal subcortical circuits (frontal cortex – striatum – globus pallidus and substantia nigra – thalamus), known to be involved in motivated behaviour, inhibitory control and social cognition (Kemp et al., 2013; Lisiecka et al., 2012; Tekin & Cummings, 2002; Utter & Basso, 2008). A specific involvement of the putamen, together with the insula, in the perception of disgust was found in a case study (Calder, Keane, Manes, Antoun, & Young, 2000). However, the patient described by Calder et al. (2000) had damage in the putamen in the left hemisphere, while the current study found the right putamen to be related to disgust perception. As we cannot exclude a left hemispheric involvement in emotion recognition because of the lower lesion coverage in the left hemisphere, the results of the current study combined with the case study of Calder et al. (2000) could possibly point to a symmetrical involvement of the putamen in disgust perception. Remarkably, in contrast with the abovementioned case study of Calder et al. (2000), we did not find a significant relation between disgust perception and insular damage. This finding is instead more in keeping with the results of a case study of Straube et al. (2010), in which no deficit in disgust perception was found in a patient with a lesion in the right insular cortex. As described earlier, we did find significant lesion–symptom relations between the insula and overall emotion recognition. Therefore, our results might indicate that the insula is involved in more general emotional processes and emotion perception, but not specifically in the perception of disgust.

Lastly, a poorer recognition of sadness was related to damage in the opercular part of the inferior frontal gyrus, which is part of the prefrontal cortex. The inferior frontal gyrus has previously been found to be related to the emotional expression of music, including sad expressions (Tabei, 2015). Furthermore, our results are in line with findings of Dal Monte et al. (2013), who found associations between damage to the anterior parts of the prefrontal cortex and a worse ability to recognize negative emotions. Our results indicate that, amongst the different negative emotions, particularly sadness might be related to this prefrontal area.

No significant lesion–symptom relations were found for the recognition of surprise and fear. In the study of Mattavelli et al. (2019), a significant relation was found between surprise recognition and the left middle frontal gyrus. In the current study, few patients had lesions in the left middle frontal gyrus. Hence, this region was not taken into account in the VLSM, which could explain the absence of a significant lesion–symptom association for surprise recognition. The absence of significant lesion–symptom relations for fear recognition, despite our finding that patients as a group were significantly worse than healthy controls in recognizing fear, could possibly be explained by findings of a recent study by McFadyen, Mattingley, and Garrido (2019), who found that the fibre density of the pathway from the right pulvinar to the amygdala was related to the ability to recognize fear. Elaborating on their findings, patients in the current study might have had a decreased fibre density in this white matter pathway, which could not be picked‐up in the lesion analysis, but which did cause fear‐recognition problems. Future studies should be performed using connectivity analyses, such as DTI, to further clarify this issue. In addition, the absence of lesion–symptom associations for fear recognition could also be the result of the relatively low lesion frequency in deep brain structures, such as the amygdala. The amygdala has frequently been described as an important structure in recognizing and processing fear (Broks et al., 1998; Lindquist et al., 2012).

A limitation of a VLSM analysis is that only damaged brain regions involved in the lesion overlap can be used for the analysis, meaning that conclusions can only be based on these involved brain regions. For instance, as described above, we cannot exclude the possibility that the left hemisphere is involved in emotion recognition, because the lesion coverage in the left hemisphere was lower than in the right hemisphere. Consequently, fewer voxels in the left hemisphere were included in the analysis, resulting in lower power for identifying significant lesion–symptom associations in the left hemisphere. Accordingly, areas with most lesion coverage (right fronto‐temporal region in this study) have the highest power for detecting lesion–symptom associations. Furthermore, the results do indicate the relative contribution of a particular brain region over another for a particular emotion, but a note of caution is due with respect to the anatomical specificity for the different emotions.

To conclude, we suggest that the architecture of the brain for emotion recognition involves a general network in the fronto‐temporal region with separate nodes related to specific basic emotions, probably with many interconnections. The insula was found to play a central role in the general network. Our results provide insight in the neural underpinnings of emotion recognition in ischaemic stroke patients. This could help in understanding how lesions in certain brain regions can selectively affect the recognition of particular basic emotions, which may in turn be related to particular behavioural disturbances. Future studies, using connectivity analyses, should be performed to complement our findings.

## Funding

This work was supported by a European Research Council (ERC) Advanced Grant (#339374) to E. H. F. de Haan.

## Conflicts of interest

All authors declare no conflict of interest.

## Author contributions

Nils S. van den Berg (Conceptualization; Data curation; Formal analysis; Investigation; Methodology; Visualization; Writing – original draft; Writing – review & editing) Edward H.F. de Haan (Conceptualization; Funding acquisition; Supervision; Writing – review & editing) Rients B. Huitema (Conceptualization; Supervision; Writing – review & editing) Jacoba M. Spikman (Conceptualization; Supervision; Writing – review & editing).

## Data Availability

The data that support the findings of this study are available from the corresponding author upon reasonable request.
